# Investigation of wine fermentation of three-leaf cayratia ( *Cayratia trifolia* L.) using *Saccharomyces cerevisiae* 2.1

**DOI:** 10.12688/f1000research.129075.4

**Published:** 2025-02-27

**Authors:** Doan Thi Kieu Tien, Le Doan Quoc Binh, Huynh Thi Ngoc Mi, Nguyen Ngoc Thanh, Bui Hoang Dang Long, Ngo Thi Phuong Dung, Ha Thanh Toan, Huynh Xuan Phong

**Affiliations:** 1 Faculty of Biological, Chemical and Food Technology, Can Tho University of Technology, Can Tho City, Vietnam; 2 Institute of Food and Biotechnology, Can Tho University, Can Tho City, Vietnam

**Keywords:** Cayratia trifolia, ethanol fermentation, Saccharomyces cerevesiae, three-leaf cayratia wine

## Abstract

**Background:**

*Cayratia trifolia* has been extensively studied for its bioactive components and medicinal properties. This study was carried out to evaluate the fermentation ability of *Saccharomyces cerevisiae* 2.1 yeast to determine suitable fermentation conditions.

**Methods:**

The suitable initial sugar content, temperature, and incubation time were investigated in the fermentation in the 100-milliliter flask scale. Besides, a trial fermentation at the 1-liter scale was conducted to assess yeast performance.

**Results:**

All treatments showed that the total sugar content decreased after 9 days of fermentation compared to the initial level. Temperature during fermentation has a direct effect on yeast activity. Temperature increases the growth of yeast and the speed of enzyme activity. The fermentation time changes depending on temperature, initial soluble solid contents, and yeast strains, leading to changes in the ethanol content after the end of fermentation. The results showed that the *S. cerevisiae* 2.1 was able to ferment at room temperature with an initial pH of 4.5, 24 °Brix, and a yeast density of 107 cells/mL. The appropriate fermentation conditions determined for *S. cerevisiae* 2.1 were 24 °Brix and incubation at room temperature (28-33°C) with a fermentation time of 11 days. In 1-L scale fermentation, three-leaf cayratia wine had 9.46% (v/v) and the fermentation efficiency was 90.34%. The wine produced had a unique color, flavor, and aroma that met the sensory evaluation of the Vietnamese national standard TCVN-3217:79.

**Concusion:**

The results showed that the *S. cerevisiae* 2.1 was able to produce wine from *Cayratia trifolia* extract in suitable conditions with high ethanol content and organoleptic values. The results of this study supplied needed information for further studies on analysis of bioactive compounds produced by the fermentation.

## 1. Introduction

In moderate consumption, wine, a traditional product of microbial fermentation, is proven to contribute benefits to human health and contains various bioactive ingredients. In the past, France and Italy were the cradles of the wine industry. Countries like Australia and New Zealand have created wine brands with modern technology and high quality. In the Asian market, particularly in Vietnam, wine has been conquering the consumer thanks to its abundant production, competitive price, and novel taste. Wines are fermented alcoholic beverages made from various base ingredients, such as apple, banana, papaya, mango, apricot, pineapple, and jackfruit juice. These are classified as grape wine, fruit wine, berry wine, vegetable wine, plant wine, and raisin wine, as well as flavors from flowers and herbs. Typical wine, natural wines (9–14% alcohol), or dessert and appetizer wines (15–21% alcohol) contain ethyl alcohol, sugar, acids, higher alcohols, tannins, aldehydes, esters, amino acids, minerals, vitamins, anthocyanins ( Amerine *et al*., 1980).

Three-leaf cayratia ( *Cayratia trifolia*) has been extensively studied for its bioactive components and medicinal properties. In particular, the abundant source distributed throughout the Mekong Delta of Vietnam gives the advantage for wine fermentation. Interestingly, *C. trifolia* antioxidant and polyphenolic compound contents were not significantly changed after fermentation ( Doan *et al*., 2018d). Additionally, its color and flavor are specific to three-leaf cayratia wine, particularly proven in our previous studies ( Doan *et al*., 2018d, 2019b), and *C. trifolia* berries are becoming an expected source of winemaking materials from grapes in Vietnam. However, the isolated thermotolerant yeast from *C. trifolia* berries is mainly used to ferment three-leaf cayratia wine. At the same time, non-thermotolerant *S. cerevisiae* is the primary source for winemaking at room temperature.

Currently, several studies were conducted for the fermentation of three-leaf *Cayratia* juice by *S. cerevisiae* which demonstrate for potential of wine fermentation using this fruit ( Doan *et al.*, 2022, 2024). Besides, yeast fermentation was proven to improve the effects of bio-active compounds including antioxidants in *C. trifolia* juice ( Doan *et al.*, 2022) by *S. cerevisiae*. According to Ngo *et al*. (2005), 50 yeast strains have been isolated from wine yeast starters in the Mekong Delta, of which *S. cerevisiae* 2.1 has the highest performance in fermentation. With these findings, the application of *S. cerevisiae 2.1* in *C. trifolia* juice fermentation promises to create a wine with positive characteristics and quality.

Therefore, this study aimed to investigate and evaluate the wine fermentation of three-leaf cayratia juice by *S. cerevisiae* isolated from rice wine starters. In addition, 1 liter of *C. trifolia* juice was fermented to complete the larger-scale fermentation process of three-leaf cayratia wine.

## 2. Methods

### 2.1 Materials

Wine fermentation of three-leaf Cayratia ( *C. trifolia* L.) using *S. cerevisiae* 2.1 was conducted between April 12 ^th^, 2022, and October 25 ^th^, 2022. Three-leaf cayratia samples were collected from shiny ripe, dark black, and undamaged berries in the Mekong Delta, Vietnam. The fresh berries were brought to the Laboratory of the Industrial Microbiology at the Institute of Food and Biotechnology, Can Tho University. The selected un-crushed ripe samples were washed several times with tap water, rinsed with distilled water, and squeezed out of flesh into juice to conduct further experiments.

*S. cerevisiae* 2.1 strain was isolated from a rice wine starter and stored at the Laboratory of Industrial Microbiology at the Institute of Food and Biotechnology Institute, Can Tho University ( Ngo *et al*., 2005). A single colony of yeast was inoculated in Yeast extract - Peptone - Dextrose (YPD broth) (yeast extract 0.5%, peptone 0.5%, D-glucose 2.0%; sterilized at 121°C for 15 min) and shaken at 180 rpm at 30°C to 10 ^9^ cells/mL of yeast inoculum level (using Haemocytometer – Neubauer).

### 2.2 Determination methods of three-leaf cayratia fermentation

**2.2.1 Determination of initial sugar for three-leaf cayratia fermentation**

Three-leaf cayratia juice was added with NaHSO _3_ (140 mg/L) and left alone for 2 h after being adjusted to 20, 24, and 28 °Brix (using sucrose provided by Bien Hoa Sugar Joint Stock Company) and pH 4.5 ( Doan *et al*., 2018b, 2018d). One mL of *S. cerevisiae* was inoculated to 99 mL of three-leaf cayratia juice (10 ^7^ cells/mL after inoculation) in a 250 mL Erlenmeyer flask. The flask was sealed with a water lock and incubated at room temperature (in triplicate). The changes in pH, °Brix, and ethanol contents were measured after 9 days of fermentation Nguyen (2007). The total sugar content of the fermentation process was analyzed according to Nielsen (2010), and fermentation efficiency was calculated.

Fermentation efficiency is calculated according to ethanol fermentation efficiency on the amount of sugar used, H (%) = (Et°*0.789*10)/(S*0.5111) where H: fermentation efficiency; Et°: alcohol content obtained (% v/v); 0.789: density of ethanol (0.789 g/cm ^3^); 10: conversion factor; S: the actual amount of sugar used in glucose (following the phenol-sulfuric method); 0.5111: the grams of theoretical ethanol obtained from 1 gram of glucose *.*

**2.2.2 Determination of temperature for three-leaf cayratia fermentation**

Three-leaf cayratia juice was prepared with °Brix, selected from Section 2.2.1. After inoculation with 1 mL yeast culture, three-leaf cayratia juice was fermented at 25°C, room temperature (28–33°C), and 35°C for 9 days. The changes in pH, °Brix, and ethanol contents were determined as described above.

**2.2.3 Determination of incubation time for three-leaf cayratia fermentation**

Three-leaf cayratia juice was prepared with °Brix selected from the experiment as mentioned in Section 2.2.1, and incubated at the selected temperature from the experiment in Section 2.2.2, on days 7, 9, 11, and 13. The changes in pH, °Brix, and ethanol content were determined (see Section 2.2.1).

**2.2.4 Trial fermentation in the 1-liter scale of the three-leaf cayratia wine**

The initial sugar content, fermentation temperature, and fermentation time were determined based on the selective results of previous screening tests to ferment 1 liter of three-leaf cayratia juice (triplicate). Sensory evaluation of wine was based on the criteria of clarity, color, aroma, taste, and overall confidence according to Vietnamese National Standard 3217:79 (TCVN 3217:79), which is done through a sensory board consisting of 10 members at the Institute of Food and Biotechnology, Institute Can Tho University. The changes in pH, °Brix, ethanol content, and total sugar content were determined (see Section 2.2.1). The chemical and microbiological criteria were analyzed by the National Agro-Forestry Fisheries Quality Assurance Center - Branch 6 (Vietnam) following Vietnam National Standard QCVN 6-3:2010/BYT.

**2.2.5 Statistical analysis**

The analyzed data were processed using Excel 2013 (RRID:SCR_016137) (Microsoft Inc., USA). The variance and the Lease Significant Difference (LSD) were analyzed using SPSS Version 27.0.1.0 (RRID:SCR_002865) and Statgraphics Centurion XV - https://www.statgraphics.com/ (Manugistics Inc., USA).

## 3. Results

### 3.1 The initial sugar of the three-leaf cayratia fermentation using *S. cerevisiae* 2.1

Three-leaf cayratia fermentation processes were conducted with an initial pH of 4.5 and an initial sugar content of 20, 24, and 28 °Brix, respectively. As to the results, all treatments show that the total sugar contents decreased after 9 days of fermentation compared to the initial level ( Table 1). The ethanol content was the highest at 7.17% v/v in the 24 °Brix treatment, which is a significant difference compared to the 20 and 28 °Brix treatments. When the sugar concentration is too high, yeast fermentation is limited due to high osmotic pressure, lowering the alcoholic production ( Kayikci & Nelsen, 2015). In principle, the rate of fermentation and the maximum amount of ethanol decreased when the sugar concentration was increased. However, a particular type of yeast strain can be selected, conditioned to grow at higher sugar concentrations (>30% sugar), and adapted ( Matei & Kosseva, 2017). In contrast, at a lower pre-fermentation Brix concentration (20°Brix), ethanol production was limited at 6.23% (v/v) which may be due to the limit in carbon sources supply insufficient nutrients for yeast growth and fermentation ( Timmermans et al., 2022).

**Table 1.** The effects of °Brix on the fermentation ability of *S. cerevisiae* 2.1.

Initial °Brix	Initial sugar content (g/L)	Used sugar content (g/L)	Residual sugar content (g/L)	Fermentation efficiency (%)	Ethanol content (% v/v) at 20°C
20	142.23	116.67	25.56	82.47 ^b^	6.23 ^b^
24	187.98	121.63	66.35	91.00 ^a^	7.17 ^a^
28	229.47	115.60	113.87	75.13 ^c^	5.64 ^b^

Note: The values in this table were the mean values of triplication. The values in a group with the same superscripted letters were not significantly different at the 95% confidence level (p < 0.05).

The total sugar content of the samples was determined using the phenol-sulfuric acid method ( Nielsen, 2010), measured at a wavelength of λ = 490 nm. The linear regression equation was y = 0.0095x + 0.0049. The total sugar content after fermentation reflected the fermentation efficiency of *S. cerevisiae* 2.1. The 24 °Brix treatment had the highest fermentation conversion efficiency of 91.00% produced by *S. cerevisiae* 2.1, 82.47% at 20 °Brix treatment, and 75.13% at 28°Brix treatment, which was significantly different from 5% (p<0.05). The fermentation conversion efficiency was proportional to the ethanol content ( Table 2). The highest alcohol content (7.17%) was produced in the 24 °Brix treatment. *S. cerevisiae* strains are tolerant to low pH, high sugar content, and high ethanol concentrations compared to other species, thus reducing the risk of bacterial contamination during industrial fermentation ( Nevoigt, 2008).

**Table 2.** The effects of incubation time on fermentation.

Temperature (°C)	Initial °Brix	°Brix after fermentation	Ethanol content (% v/v) at 20°C
Room temp. (28–33°C)	24	12.17	6.94 ^a^
25	24	14.17	6.16 ^b^
35	24	16.83	4.28 ^c^

Note: The values in this table were the mean values of triplication. The values in a group with the same superscripted letters were not significantly different at the 95% confidence level (p < 0.05).

### 3.2 Temperature for three-leaf cayratia fermentation

The ethanol content was significantly different depending on the incubation temperature ( Doan *et al.*, 2019a). Temperature during fermentation has a direct effect on yeast activity. In this study, the ethanol content was produced at 6.94% (v/v) at room temperature, higher than that of 25°C (6.16% v/v) and 35°C (4.28% v/v). Based on a study, the optimum temperature for the growth and multiplication of *S. cerevisiae* is 30-35°C and is usually inhibited at temperatures higher than 39.8°C ( Walsh & Martin, 1977).

Temperature increases the growth of yeast and the speed of enzyme activity. Because of the increased membrane fluidity, cell sensitivity to the toxic effects of alcohol increases with temperature. Thus, yeast viability may rapidly decline at temperatures above 20°C during wine fermentation. Normal cider production occurs at 20–25°C and lasts for 1 to 4 weeks ( Waites *et al*., 2001).

### 3.3 Fermentation time for three-leaf cayratia wine

The fermentation time changes depending on temperature, initial soluble solid contents, and yeast strains, leading to changes in the ethanol content after the end of fermentation. The results showed the highest ethanol content at 11 days compared to 13, 9, and 7 days with 8.93%, 8.54%, 6.94%, and 6.23% v/v, respectively. There were no significant differences between 7 to 9 days and 11 to 13. However, these values were significantly different between days 7 and 11 ( Table 3).

**Table 3.** The fermentation temperature results of *S. cerevisiae* 2.1.

Incubation time (day)	Initial °Brix	°Brix after fermentation	Ethanol content (% v/v) at 20°C
7	24	14.17	6.94 ^b^
9	24	12.50	6.23 ^b^
11	24	9.33	8.93 ^a^
13	24	9.33	8.54 ^a^

Note: The values in this table were the mean values of triplication. The values in a group with the same superscripted letters were not significantly different at the 95% confidence level (p < 0.05).

The end time of fermentation was inversely proportional to the degree of Brix after fermentation. Table 3 indicated the higher ethanol concentration of 8.54 % (v/v) and lower Brix of 9.33°Brix at the longest fermentation time at 13 days compared to the shorter times. The longer the time, the lower the °Brix, and the higher the ethanol concentration produced. Initially, when the ethanol concentration was quite low, the ethanol concentration began to increase, and the sugar content decreased.

### 3.4 Trial fermentation in the 1-liter scale of the three-leaf cayratia wine

The fermentation of fruit wine was tested with a volume of 1 liter, surveyed with a density of *S. cerevisiae* 2.1 at 10 ^7^ cells/mL for 11 days at room temperature, and the juice was adjusted to pH 4.5 and 24 °Brix. From the above experiments, it can be seen that *S. cerevisiae* 2.1 produced an ethanol content of 9.49% (v/v), and the fermentation efficiency was quite high, reaching 90.34%.

**Figure 1.**
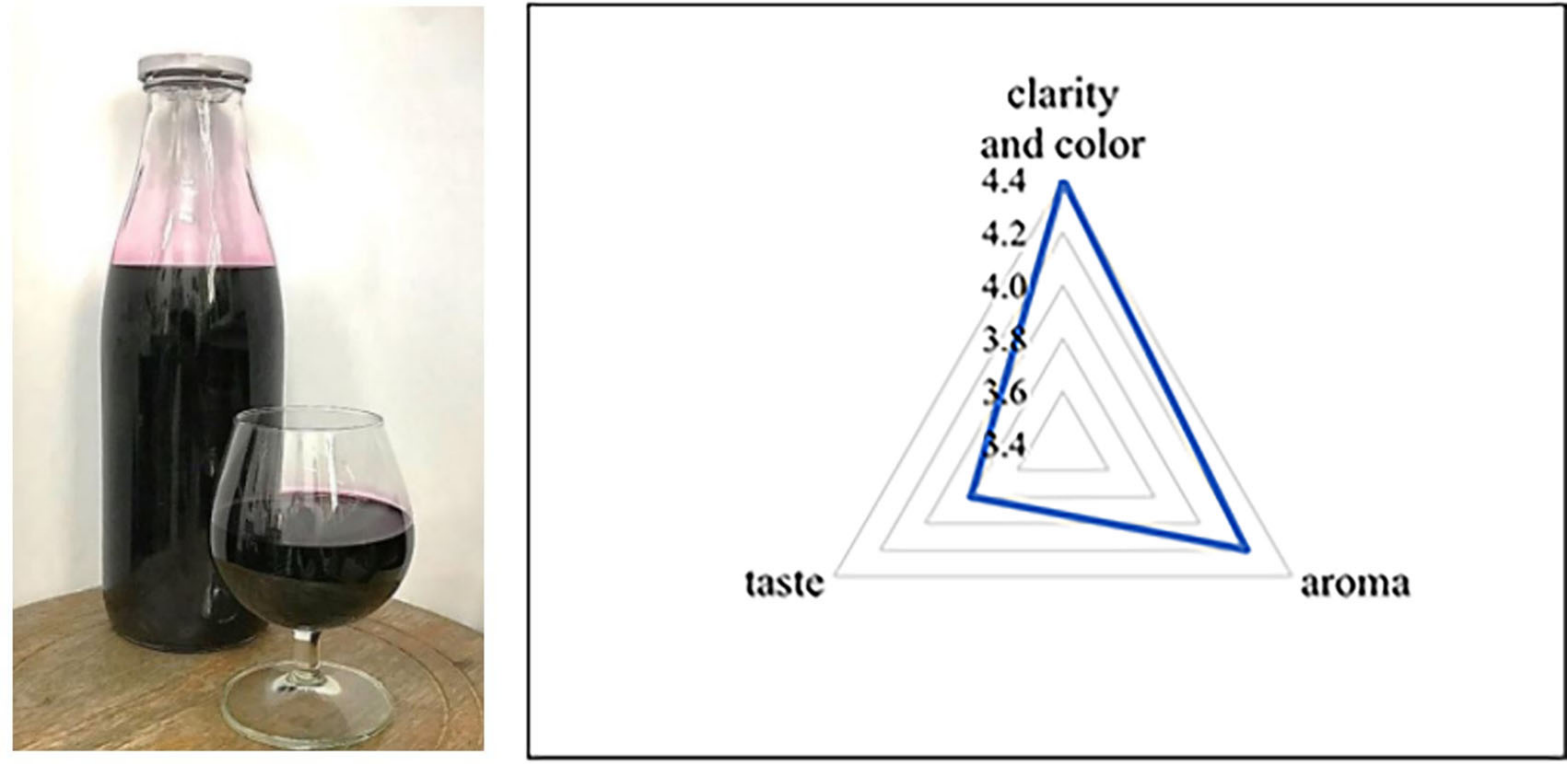
Three-leaf cayratia wine and sensory evaluation chart.

After fermentation, the sensory properties were evaluated according to the criteria of TCVN 3217:79. Three-leaf wine was assessed as good in clarity and color, aroma, and taste, reaching 4.4, 4.2, and 3.8 points, respectively ( Figure 1). Sensory evaluation results show that the wine had no strange, cloudy smell, and the color and odor were specific to the product. Compared to the TCVN 3217:79, the sensory quality assessment of left-sided wine received a good rating.

The chemical and microbiological criteria of the final product were analyzed by the National Agro-Forestry Fisheries Quality Assurance Center - Branch 6, Vietnam. The results are shown in Table 4. These values are met to the Vietnam National Standard QCVN 6-3:2010/BYT.

**Table 4.** Quality evaluation of cayratia wine.

Criteria	Vietnam National Standard QCVN 6-3:2010/BYT	Method	Result
Ethanol (% v/v)		Ref.AOAC 972.11 (LOQ = 0.025%)	9.49
Methanol (mg/L)	<400	Ref.AOAC 972.11 (LOQ = 0.025%)	n.d.
Total microaerophile (CFU/mL)	1,000	ISO 4833-1:2013	<10
Total yeast and mold (CFU/mL)	100	ISO 215227-2:2008	<10
Coliforms (CFU/mL)	0	ISO 4832:2006	0
*E. coli* (CFU/mL)	0	ISO 16649-2:2001	0
Fecal Streptococci (CFU/mL)	0	ISO 7899-2:2000	0
*Clostridium perfringens* (CFU/mL)	0	ISO 7937:2004	0

These parameters were analyzed by National Agro-Forestry Fisheries Quality Assurance Center - Branch 6, Vietnam. n.d.: not detected.

## 4. Discussion

In a previous study, we isolated 151 strains of yeast from 53 samples of *C. trifolia* in the Mekong Delta. Of these, 30 isolates were highly fermentative and produced ethanol concentrations between 6.0 and 9.9% (v/v) ( Doan *et al*., 2018c). The fermentation ability of thermotolerant yeast isolates from three-leaf juice showed that the survey with 3.6 of the natural pH, 22 °Brix by adding sucrose, the initial yeast density of 10 ^6^ cells/mL, and 7 days of incubation time at 37°C. *Saccharomyces* sp. CT3.2 has a fast-filling time for the gas column in the Durham tube (after 12 h), and the ethanol content after fermentation was not high (5.4% v/v). In contrast, *Saccharomyces* sp. HG1.3 has a slower gas column filling time (after 18 h) but produces the highest ethanol content (9.9% v/v) ( Doan *et al*., 2018b). Meanwhile, with initial fermentation conditions of pH 4.5, and 20 °Brix fermented at 35°C for 6 days, *Saccharomyces* sp. HG1.3 gave the ethanol content of post-fermentation three-leaf fruit wine 12.0% v/v ( Doan *et al*., 2018d).

The fermentation ability of thermotolerant yeasts was investigated at pH 4.5, 22 °Brix, 10 ^7^ cells/mL of the initial three-leaf juice, and at 37°C for 7 days. The ethanol contents of three-leaf wine produced by CM3.2, CM3.3, and BT1.2 were 8.95%, 7.01%, and 6.79% v/v, respectively ( Torija *et al.*, 2003; Doan *et al*., 2018a).

The fermentation of 1-liter results shows that the ethanol content met the Vietnam standard 6-3:2010/BYT of the Ministry of Health for wine ( Vietnam National Standard 3217:79, 1979), which is also similar to fermentation studies on watermelon wine ( Nguyen, 2010; Ngo *et al*., 2011), pineapple wine ( Nguyen *et al*., 2013), and sim wine ( Nguyen, 2010). However, the concentration value is lower than that of fruit wine fermented by a tolerant yeast strain isolated from fruit but aged at 35°C; for example, the strain *S. cerevisiae* AG2.1 is 11.36% (v/v) ( Doan *et al*., 2018c), and *S. cerevisiae* CM3.2 is 12.46% v/v ( Doan *et al*., 2019b).

The fermentation of 1 L results shows that the ethanol content meets the Vietnam Standard 6-3:2010/BYT of the Ministry of Health for wine, which is also similar to fermentation studies on watermelon wine ( Ngo *et al*., 2011), pineapple wine ( Nguyen *et al*., 2013), and sim wine ( Nguyen, 2010). However, the concentration value is lower than that of fruit wine fermented by a tolerant yeast strain isolated from fruit but aged at 35 °C; for example, the strain *S. cerevisiae* AG2.1 is 11.36% (v/v) ( Doan *et al*., 2018c), *S. cerevisiae* CM3.2 is 12.46% v/v ( Doan *et al*., 2019b).

## 5. Conclusion

This study aimed to test the fermentative capacity of the *S. cerevisiae* 2.1 strain isolated from a starter on three-leaf cayratia juice. However, microbiological and physicochemical criteria have not yet been implemented. The results showed that the *S. cerevisiae* 2.1 strain was able to ferment at room temperature with an initial pH of 4.5, 24 °Brix, and yeast density of 10 ^7^ cells/mL. Preliminary 1 liter of fermentation for 11 days produced an ethanol content of 9.49% (v/v). The chemical and microbiological criteria of final product was met to the Vietnam National Standard QCVN 6-3:2010/BYT.

### 5.1 Ethical considerations

This study was approved by the research group at Can Tho University and Can Tho University of Technology, Vietanm. All authors mentioned in the manuscript have agreed to authorship, read and approved the manuscript, and provided consent for submission and subsequent publication of the manuscript. The authors declare no conflicts of interest.

## Data Availability

Figshare: Wine fermentation of three-leaf cayratia. figshare. Dataset, https://doi.org/10.6084/m9.figshare.22256188.v1 ( Phong, 2023). Data are available under the terms of the Creative Commons Attribution 4.0 International license (CC-BY 4.0).
