# Supplementary material for: Investigation of wine fermentation of three-leaf cayratia ( Cayratia trifolia L.) using Saccharomyces cerevisiae 2.1
Source: F1000Res. 2025 Sep 23;12:312. Originally published 2023 Mar 21. [Version 5] doi: 10.12688/f1000research.129075.5 (PMC12417979; doi:10.12688/f1000research.129075.5)
Supplement: Peer review history

# Reviewer report: Reviewer response for version 5

**DOI:** 10.5256/f1000research.188140.r416786

**Published:** 2025-09-27

**Authors:** Susan Abdul Raheem Hasan

**Affiliations:** 1 Biology, University of Baghdad, Baghdad, Iraq

No further comments

Is the work clearly and accurately presented and does it cite the current literature?

Yes

If applicable, is the statistical analysis and its interpretation appropriate?

I cannot comment. A qualified statistician is required.

Are all the source data underlying the results available to ensure full reproducibility?

Yes

Is the study design appropriate and is the work technically sound?

Yes

Are the conclusions drawn adequately supported by the results?

Partly

Are sufficient details of methods and analysis provided to allow replication by others?

Yes

Reviewer Expertise:

Microbiologist and expert in biological fermentation processes, with a particular focus on probiotics and plants.

I confirm that I have read this submission and believe that I have an appropriate level of expertise to confirm that it is of an acceptable scientific standard.

# Reviewer report: Reviewer response for version 4

**DOI:** 10.5256/f1000research.178047.r408095

**Published:** 2025-09-08

**Authors:** Nguyen Khoi Hong Nguyen

**Affiliations:** 1 Nguyen Tat Thanh University, Ho Chi Minh City, Vietnam

I appreciate the efforts of the authors in the research process, however, if the research could add the following content, it would be more complete:

 - The author's introduction also stated the purpose of the research, however, What are the benefits of *Cayratia trifolia *wine? Before and after fermentation, how have the bioactive components in *Cayratia trifolia* been published in previous studies? If the authors could add more to the overview, it would be better.

 - In my personal opinion, the author's analysis and evaluation method needs to be presented in more detail.

 - The explanation of the basis of the results is mainly based on the authors' previous research, if other studies are added, it would be more convincing.

 - The conclusion has summarized the data achieved by the topic, if 1-2 sentences about the potential use or value that the research brings are added, it would be better.

Is the work clearly and accurately presented and does it cite the current literature?

Yes

If applicable, is the statistical analysis and its interpretation appropriate?

Partly

Are all the source data underlying the results available to ensure full reproducibility?

Yes

Is the study design appropriate and is the work technically sound?

Yes

Are the conclusions drawn adequately supported by the results?

Yes

Are sufficient details of methods and analysis provided to allow replication by others?

Yes

Reviewer Expertise:

My area of expertise is food technology, particularly in processing sectors such as seafood, meat, fruits and vegetables, fermentation, and preservation technology. In my assessment, the content of this study is acceptable, as Cayratia trifolia represents a novel plant source. The research provides a dataset that could serve as a valuable reference foundation for future studies.

I confirm that I have read this submission and believe that I have an appropriate level of expertise to confirm that it is of an acceptable scientific standard.

# Response

**Published:** 2025-09-13

**Authors:** Huynh Xuan Phong

**Affiliations:** Can Tho University, Vietnam

On behalf of the authors, I would like to express our sincere gratitude to the Reviewer for the valuable comments.

**1. The author's introduction also stated the purpose of the research, however, what are the benefits of *Cayratia trifolia* wine? Before and after fermentation, how have the bioactive components in *Cayratia trifolia* been published in previous studies? If the authors could add more to the overview, it would be better.**

 Thank you for your suggestions. The benefits from wine are demonstrated by many previous papers [Kumar et al., 2011; Rousdy et al., 2025] but we do not have the benefits from *Cayratia trifolia* wine directly. However, our previous study reported the bioactive compound from the *Cayratia trifolia* juice before and after fermentation [Doan et al., 2018d]. We add some more information in the Introduction and the source of these articles in the Reference.

**2. In my personal opinion, the author's analysis and evaluation method needs to be presented in more detail.**

 Thank you for your comments. We added more details on the analysis and evaluation methods in a new section (Section 2.2.5) with references. We hope the information can provide a better understanding to the viewer and for the study.

**3. The explanation of the basis of the results is mainly based on the authors' previous research, if other studies are added, it would be more convincing.**

 This is a great suggestion. We edit the discussion with added information on the results from previous research and other studies. We hope this can improve the quality of the discussion.

**4. The conclusion has summarized the data achieved by the topic, if 1-2 sentences about the potential use or value that the research brings are added, it would be better.**

 Thanks for your comments. We edited the conclusion, which includes information on the value and contribution of applying *S. cerevisiae* 2.1 to the production of three-leaf caraytia wine.

# Reviewer report: Reviewer response for version 4

**DOI:** 10.5256/f1000research.178047.r408091

**Published:** 2025-09-02

**Authors:** Shatha Abdullah Allaith

**Affiliations:** 1 University of Kerbala Faculty of Agriculture (Ringgold ID: 125663), Kerbala, Karbala Governorate, Iraq

1. The experimental design is not up to the required scientific standard, especially since it used a yeast isolate from a previous study but did not utilize it well to demonstrate the importance of the current study.

 2. In paragraph 2.1, the researcher did not cite any references when using the YPD broth medium. Why? Why did he use a cell concentration of 10^9 at a temperature of 30°C without citing any references?

 3. In paragraph 2.2.1, he relied solely on fermentation efficiency. It would have been preferable to study fermentation kinetics using a mathematical model, such as RSM or others, to study the economics of fermentation so that it could be applied commercially.

 4. When measuring the optimum conditions for wine production, it would have been preferable to add it to the results of the previous research from which the yeast was isolated or to include it in a new study that reflects the scientific content of the topic and title of the research. 

 5. It would have been preferable for the statistical analysis to rely on the mean ± experimental error, as this provides a clearer explanation of the standard error of the experimental parameters.

 6. Why did the researcher rely on Brix in the experiment without specifying the type of device used to measure which concentrations, and why? Especially since it is widely used in the fermentation industry, which includes sugar.

 7. In Table 1, were the initial sugar content and residual sugar content estimated using the phenolsulfuric acid method? The method was not explained in the method.

 8. In Table 2, how the ethanol content was calculated each time at 20°C is shown, while the optimal temperature used was room temperature. The ethanol content was not explained as to how it was calculated each time.

 9. The discussion is very brief and unclear, and the research results were not compared well with other studies.

 10. The conclusion did not mention the challenges and obstacles and finding appropriate solutions for them in the wine industry, despite the difficulties facing the fermentation process, nor did it mention how conditions are controlled at the laboratory level and transformed to a commercial scale.

 I confirm that I have read this manuscript carefully, and based on my experience in the field of food technology and industrial fermentation, I decline to accept the research for the reasons stated above. I hope the researcher will reconsider the above points to improve the manuscript in the future and experimental error, as this provides a clearer explanation of the standard error of the experimental parameters.

Is the work clearly and accurately presented and does it cite the current literature?

Yes

If applicable, is the statistical analysis and its interpretation appropriate?

Partly

Are all the source data underlying the results available to ensure full reproducibility?

Partly

Is the study design appropriate and is the work technically sound?

Partly

Are the conclusions drawn adequately supported by the results?

Partly

Are sufficient details of methods and analysis provided to allow replication by others?

No

Reviewer Expertise:

food technology and industrial fermentation

I confirm that I have read this submission and believe that I have an appropriate level of expertise to state that I do not consider it to be of an acceptable scientific standard, for reasons outlined above.

# Response

**Published:** 2025-09-13

**Authors:** Huynh Xuan Phong

**Affiliations:** Can Tho University, Vietnam

On behalf of the authors, I would like to express our sincere gratitude to the Reviewer for the valuable comments. The referee’s comments were very useful in improving the quality and readability of our manuscript. We have followed the comments and made revisions carefully, point by point.

**1. The experimental design is not up to the required scientific standard, especially since it used a yeast isolate from a previous study but did not utilize it well to demonstrate the importance of the current study.**

 Thank you for your comments. We have carefully revised the experimental design and method description. We also provide a more comprehensive discussion with additional references to strengthen the argument. We hope the revision helps improve the quality of this study.

**2. In paragraph 2.1, the researcher did not cite any references when using the YPD broth medium. Why? Why did he use a cell concentration of 10^9 at a temperature of 30°C without citing any references?**

 Thank you for your comments. We use the protocol applied in a previous study of our group (ref: 10.22144/ctujos.2024.372). We modified the text that supplies the reference to provide clear information on the protocol applied in this study.

**3. In paragraph 2.2.1, he relied solely on fermentation efficiency. It would have been preferable to study fermentation kinetics using a mathematical model, such as RSM or others, to study the economics of fermentation so that it could be applied commercially.**

 In the scope of this study, we intended to conduct a trial fermentation on a laboratory scale. The focus of this study was to investigate the properties of fermentation products. The optimization by RSM should be conducted in further studies on a larger scale, which can be more suitable for scale-up practical fermentation in industries. However, we added a recommendation in the section for further studies to conduct RSM analysis for optimization of the conditions.

**4. When measuring the optimum conditions for wine production, it would have been preferable to add it to the results of the previous research from which the yeast was isolated or to include it in a new study that reflects the scientific content of the topic and title of the research.**

 This is a valuable comment. We added the discussion on the results of the previous studies on *S. cerevisiae *2.1 in comparison with this study in the discussion section. This can clearly help improve the scientific content of this research.

**5. It would have been preferable for the statistical analysis to rely on the mean ± experimental error, as this provides a clearer explanation of the standard error of the experimental parameters.**

 We agree with your comment. we added the experimental error to all analyzed data in this study.

**6. Why did the researcher rely on Brix in the experiment without specifying the type of device used to measure which concentrations, and why? Especially since it is widely used in the fermentation industry, which includes sugar.**

 We agree on the comments that the information on the device for measuring the Brix should be included in the paper. For that, we added information on the device and the method description in Section of this study 2.2.5. Besides, the Section “2.2.5 Analytical methods” was added to describe the methods of quantification of parameters employed in this study.

**7. In Table 1, were the initial sugar content and residual sugar content estimated using the phenolsulfuric acid method? The method was not explained in the method.**

 This is a great recommendation. We added information on the phenol-sulfuric methods with a reference. Besides, we described the method in detail in section 2.2.5.

**8. In Table 2, how the ethanol content was calculated each time at 20°C is shown, while the optimal temperature used was room temperature. The ethanol content was not explained as to how it was calculated each time.**

 The ethanol at 20°C was calculated by using the correction table. For that, measured ethanol contents at room temperature were converted to equivalent ethanol contents at 20°C. We added information on the ethanol exchanging method in Section 2.2.5 with a reference.

**9. The discussion is very brief and unclear, and the research results were not compared well with other studies.**

 Thank you for your suggestion. We added new discussions in Section 4 to compare the obtained results with previous studies on the related topic. Besides, we add some recommendations for further studies to improve the practical application of the study. We hope that the discussion can help gain a better understanding and improve the quality of the paper on the results of this study.

**10. The conclusion did not mention the challenges and obstacles and finding appropriate solutions for them in the wine industry, despite the difficulties facing the fermentation process, nor did it mention how conditions are controlled at the laboratory level and transformed to a commercial scale.**

 This comment is great. Since we did a trial fermentation and optimization of conditions at laboratory scale. We think that further studies can conduct further optimization using the RSM method at a larger scale, such as pilot and factory scale. That can help apply the results commercially. We added a recommendation in Section 3.4.

# Reviewer report: Reviewer response for version 4

**DOI:** 10.5256/f1000research.178047.r368792

**Published:** 2025-03-17

**Authors:** Javier Alonso-Del-Real

**Affiliations:** 1 Instituto de Biomedicina de Valencia, Valencia, Spain; 2 Instituto de Agroquimica y Tecnología de Alimentos, Paterna, Spain

Although authors address some of the concerns raised before, the paper still contain some important issues that must be reviewed.

 Authors claim that moderate consumption of wine is healthy. As I pointed out in the previous round, recent evidence clearly suggest that no level of alcohol consumption is healthy. Please, correct appropriately.

 Authors still hold that temperatures over 20ºC provoke defects in cell viability, which is in strong disagreement with evidence on S. cerevisiae temperature adaptation.

 Also, authors should make the analysis of wine composition during the fermentation under the light of S. cerevisiae metabolic properties, exhaustively studied for a very long time.

 Another thing that I find interesting, is the fact that fermentation is not  conducted until dryness, and ethanol starts lowering down between days 11 and 13. Authors should discuss that.

 Please, try to avoid trivial observations such as "The longer the time, the lower the °Brix, and the higher the ethanol concentration produced. Initially, when the ethanol concentration was quite low, the ethanol concentration began to increase, and the sugar content decreased."

Is the work clearly and accurately presented and does it cite the current literature?

No

If applicable, is the statistical analysis and its interpretation appropriate?

No

Are all the source data underlying the results available to ensure full reproducibility?

No

Is the study design appropriate and is the work technically sound?

Partly

Are the conclusions drawn adequately supported by the results?

No

Are sufficient details of methods and analysis provided to allow replication by others?

No

Reviewer Expertise:

Laboratory scale wine fermentation by Saccharomyces cerevisiae

I confirm that I have read this submission and believe that I have an appropriate level of expertise to state that I do not consider it to be of an acceptable scientific standard, for reasons outlined above.

# Response

**Published:** 2025-09-13

**Authors:** Huynh Xuan Phong

**Affiliations:** Can Tho University, Vietnam

On behalf of the authors, I would like to express our sincere gratitude to the Reviewer for the valuable comments. The referee’s comments were very useful in improving the quality and readability of our manuscript. We have followed the comments and made revisions carefully, point by point.

**1. Authors claim that moderate consumption of wine is healthy. As I pointed out in the previous round, recent evidence clearly suggest that no level of alcohol consumption is healthy. Please, correct appropriately.**

 Thank you for your comment. We checked again and removed the statement that moderate wine consumption is healthy in the introduction part of this manuscript. Besides, we provide one reference [ref: https://doi.org/10.3390/foods11121804] to support the statement of bioactive compounds in wine.

**2. Authors still hold that temperatures over 20ºC provoke defects in cell viability, which is in strong disagreement with evidence on *S. cerevisiae* temperature adaptation.**

 Thank you for your comment. We agree that the statement that “20ºC reduced cell viability” is not appropriate. We removed the related statement in section 3.2 of the manuscript.

**3. Also, authors should make the analysis of wine composition during the fermentation under the light of *S. cerevisiae* metabolic properties, exhaustively studied for a very long time.**

 We understand the importance of wine composition to understand *Saccharomyces cerevisiae* fermentation. However, in the scope of this study, we analyze the composition of wine at the end of the fermentation process, which can prove the wine quality that satisfies the standard.

 We have added a suggestion in Section for further studies conducted on wine composition analysis during the fermentation process in the third paragraph of Section 4. We hope it can help readers understand more about the wine fermentation of the three-leaf caraytia.

**4. Another thing that I find interesting, is the fact that fermentation is not conducted until dryness, and ethanol starts lowering down between days 11 and 13. Authors should discuss that.**

 This is an important suggestion. We found a lower ethanol content on day 13 compared to day 11, although the difference was not significant. This can be caused by a possible ethanol assimilation process by ethanol dehydrogenase enzyme [ref: https://doi.org/10.1111/j.1567-1364.2008.00387.x]. For that, we add a discussion in Section 4 with an additional reference.

# Reviewer report: Reviewer response for version 4

**DOI:** 10.5256/f1000research.178047.r368791

**Published:** 2025-03-05

**Authors:** Susan Abdul Raheem Hasan, Muazaz Azeez Hasan AL-Hadeethi

**Affiliations:** 1 Botany, University of Baghdad, Baghdad, Baghdad, Iraq; 2 Biology, University of Baghdad, Baghdad, Iraq

I have no further comments.

Is the work clearly and accurately presented and does it cite the current literature?

Yes

If applicable, is the statistical analysis and its interpretation appropriate?

I cannot comment. A qualified statistician is required.

Are all the source data underlying the results available to ensure full reproducibility?

Yes

Is the study design appropriate and is the work technically sound?

Yes

Are the conclusions drawn adequately supported by the results?

Partly

Are sufficient details of methods and analysis provided to allow replication by others?

Yes

Reviewer Expertise:

Microbiologist and expert in biological fermentation processes, with a particular focus on probiotics and plants.

We confirm that we have read this submission and believe that we have an appropriate level of expertise to confirm that it is of an acceptable scientific standard.

# Reviewer report: Reviewer response for version 3

**DOI:** 10.5256/f1000research.163527.r289555

**Published:** 2024-06-22

**Authors:** Susan Abdul Raheem Hasan, Muazaz Azeez Hasan AL-Hadeethi

**Affiliations:** 1 Biology, University of Baghdad, Baghdad, Iraq; 2 Botany, University of Baghdad, Baghdad, Baghdad, Iraq

The article (Investigation of wine fermentation of three-leaf cayratia (Cayratia trifolia L.) using Saccharomyces cerevisiae 2.1) investigates the optimal conditions for the fermentation process. It discusses each condition and whether it was appropriate to meet the best efficiency (the aim of the study). This purpose was cleared in the context in detail. The materials were mentioned with their sources clearly and appropriate to their need and use in methods. Some methods details were not mentioned, but the source was written so the reader or researcher could follow the references for some unclear steps in the processes of this work. All following parts of the article were transparent and agreed or disagreed with the indexed sources and references for results and conclusion. It also mentioned some further criteria, such as microbiological and physiochemical to be studied. Some issues need to be solved or cleared to improve the article:

- Method in abstract missing details such as (volumes, weights, etc.) that were tested, which then were cleared in the main part of the manuscript.
- It is crucial to emphasize the need for additional Statistical analysis, which will further strengthen the validity of the findings.
- It is essential to discuss the effect of a lower amount of sugar, as this could potentially significantly impact the fermentation process.
- Further information and details should be added to a conclusion as in the abstract.

Is the work clearly and accurately presented and does it cite the current literature?

Yes

If applicable, is the statistical analysis and its interpretation appropriate?

I cannot comment. A qualified statistician is required.

Are all the source data underlying the results available to ensure full reproducibility?

Yes

Is the study design appropriate and is the work technically sound?

Yes

Are the conclusions drawn adequately supported by the results?

Partly

Are sufficient details of methods and analysis provided to allow replication by others?

Yes

Reviewer Expertise:

Microbiologist and expert in biological fermentation processes, with a particular focus on probiotics and plants.

We confirm that we have read this submission and believe that we have an appropriate level of expertise to confirm that it is of an acceptable scientific standard, however we have significant reservations, as outlined above.

## References

[1] : The fermentation conditions of low alcoholic three-leaved (Cayratia trifolia (L.) Domin) cider using Saccharomyces cerevisiae HG1.3. *Journal of Applied Biology & Biotechnology* .2024; 10.7324/JABB.2024.162329 10.7324/JABB.2024.162329

# Response

**Published:** 2025-02-07

**Authors:** Huynh Xuan Phong

**Affiliations:** Can Tho University, Vietnam

**1. Method in abstract missing details such as (volumes, weights, etc.) that were tested, which then were cleared in the main part of the manuscript.**

 This is a valuable comment. We clarified the information on the volumes of each fermentation trial (100 milliliters and 1 liter) in the abstract section.

**2. It is crucial to emphasize the need for additional Statistical analysis, which will further strengthen the validity of the findings.**

 We agree with your comments. For that, we added Section 2.2.5 on Lines 87-91 to clarify the method of statistical analysis.

**3. It is essential to discuss the effect of a lower amount of sugar, as this could potentially significantly impact the fermentation process.**

 A discussion on the effect of lower sugar amount on the fermentation was added on Lines 103-106. We also add a reference to clarify the explanation.

**4. Further information and details should be added to a conclusion as in the abstract.**

 We revised the conclusion part of the abstract to include essential findings from our study. For that, we clarify that the result of this study provides information for further investigation conducted in the analysis of bioactive compounds produced by fermentation.

# Reviewer report: Reviewer response for version 3

**DOI:** 10.5256/f1000research.163527.r263138

**Published:** 2024-05-06

**Authors:** Javier Alonso-Del-Real

**Affiliations:** 1 Instituto de Biomedicina de Valencia, Valencia, Spain; 2 Instituto de Agroquimica y Tecnología de Alimentos, Paterna, Spain

The present paper aims to describe the suitability of a particular yeast strain isolated in a previous related work for the fermentation of *Cayratia trifolia* fruits. To that purpose authors conduct several fermentation experiments with different amount of initial sugars and temperature set up. Based on that, they determined the most suitable conditions in micro scale fermentation, and then scale up to pilot scale with a positive outcome.

 Although the manuscript harbors potential interest from a biotechnological point of view, several issues need to be addressed.

 First of all, some of the conclusions presented are not supported by the results or the experiments conducted. Also, The reader may have trouble finding the link between some of the

 conclusions and the results supporting them. The authors may find these cases detailed below so that they can improve this aspect.

 Moreover, authors should clearly explain the statistical treatment of the obtained data. In the paper, it is stated that three replicates have been used, but there is no indication about what are the values presented (presumably mean values). In addition, errors or standard deviations, as well as tests conducted to determine possible significant differences were not shown.

 Also, the writing itself is an issue of concern, as it is sometimes difficult to follow. I would strongly recommend to consider a professional English editing service. Not only phrasing, but punctuation is confusing at some points. Also, the text is unnecessarily repetitive in some occasions, and ideas are not expressed in a cohesive and coherent way.

 Some particular comments reflecting these general considerations are the following:

 -In Abstract Methods section, the sentence "The temperature for three-leaf cayratia fermentation, incubation time, and preliminary 1 liter of three-leaf cayratia wine fermentation were determined" should be rephrased. Should "preliminary" be accompanying a noun?

 -In general, Abstract Methods section reflects a content more suitable for a results section. Please, modify that section accordingly.

 -The Conclusion Abstract section also has a content more suitable for a results section.

 - The first sentence of the introduction about the impact of alcoholic beverages on health is not in agreement with WHO, based on several  recent studies such as Anderson BO et al, 2023 [Ref 2] 

 -The word "fermentability" is used as a characteristic typical of yeast, but it would rather refer to a carbon source or a growth medium. Anyway, authors should provide an objective set of parameters defining quality of yeast in fermentation to justify the strain selection.

 -Some background about in vitro fermentation of three-leaf cayratia, their properties and biotechnological potential should be furthered commented. Otherwise, the work would remain quite out of focus.

 -In section 3.1, authors state that yeast die in the highest sugar concentration condition. However,  results from a cell viability test should be provided to prove it. Anyway, another explanation would be a slower fermentation rate, probably because adjustment to higher sugar concentrations at the initial must normally requires a significant amount of time. In any case, it would be nice to have data of the dynamics of both the fermentation and the yeast growth.

 -In section 3.2, authors claim that ordinary yeast optimal temperature is 30ºC, but they probably refer to S. cerevisiae. There is a wide range of optimal growth temperatures for other yeast. Also, they state that their growth is inhibited above 32ºC, but this is simply not correct [Refer Ref 1]. After that, authors affirm that temperatures above 20ºC could provoke yeast viability to lower down, which seems contradictory. All in all, discussion of the results are not appropriated in its current state.

 -In section 3.3, the relationship between fermentation and temperature is introduced. However, the results presented do not correspond to anything about temperature. Also,  the method to determine the end of the fermentation is not presented. It rather seems a serial sampling of the same fermentations at different time points.

 -Figure 1 relevance is hard to interpret.

Is the work clearly and accurately presented and does it cite the current literature?

No

If applicable, is the statistical analysis and its interpretation appropriate?

No

Are all the source data underlying the results available to ensure full reproducibility?

No

Is the study design appropriate and is the work technically sound?

Partly

Are the conclusions drawn adequately supported by the results?

No

Are sufficient details of methods and analysis provided to allow replication by others?

No

Reviewer Expertise:

Laboratory scale wine fermentation by Saccharomyces cerevisiae

I confirm that I have read this submission and believe that I have an appropriate level of expertise to state that I do not consider it to be of an acceptable scientific standard, for reasons outlined above.

## References

[1] : Temperature adaptation markedly determines evolution within the genus Saccharomyces. *Appl Environ Microbiol* .2011;77(7) : 10.1128/AEM.01861-10 2292-302 10.1128/AEM.01861-10 21317255 PMC3067424

[2] : Health and cancer risks associated with low levels of alcohol consumption. *Lancet Public Health* .2023;8(1) : 10.1016/S2468-2667(22)00317-6 e6-e7 10.1016/S2468-2667(22)00317-6 36603913 PMC9831798

# Response

**Published:** 2025-02-07

**Authors:** Huynh Xuan Phong

**Affiliations:** Can Tho University, Vietnam

**1. First of all, some of the conclusions presented are not supported by the results or the experiments conducted. Also, The reader may have trouble finding the link between some of the** **conclusions and the results supporting them. The authors may find these cases detailed below so that they can improve this aspect.**

 Thank you for your comments, we added discussion on Lines 103-106, 147-148

 In this information, we have linked the data with corresponding discussions that improve the discussion quality.

**2. Moreover, authors should clearly explain the statistical treatment of the obtained data. In the paper, it is stated that three replicates have been used, but there is no indication about what are the values presented (presumably mean values). In addition, errors or standard deviations, as well as tests conducted to determine possible significant differences were not shown.**

 This is a valuable comment, we clarified the statistical analysis methods on the information on Lines 87-91 of Section 2.2.5.

**3. Also, the writing itself is an issue of concern, as it is sometimes difficult to follow. I would strongly recommend to consider a professional English editing service. Not only phrasing, but punctuation is confusing at some points. Also, the text is unnecessarily repetitive in some occasions, and ideas are not expressed in a cohesive and coherent way.**

 Thank you for your suggestion, we have read and made needed changes to improve the English writing of the whole manuscript.

**4. In Abstract Methods section, the sentence "The temperature for three-leaf cayratia fermentation, incubation time, and preliminary 1 liter of three-leaf cayratia wine fermentation were determined" should be rephrased. Should "preliminary" be accompanying a noun?**

 We changed the word “preliminary 1-liter” into “trial fermentation in the 1-liter-scale of three-leaf cayratia wine” in the abstract section and the names of sections 2.2.4 (line 76) and 3.4 (Line 152).

**5. In general, Abstract Methods section reflects a content more suitable for a results section. Please, modify that section accordingly.**

 Thank you for your suggestion, we have modified the methods of the abstract section and included only the needed methods for the study. The information related to results was relocated to the result part of the abstract.

**6. The Conclusion Abstract section also has a content more suitable for a results section.**

 We relocated the information related to results from the conclusion to the results of the abstract section. The conclusion part is revised to include only the needed conclusion of the study. Besides, we have clarified that the results of this study can be utilized as a base for further studies conducted in exploring bioactive compounds generated from the fermentation.

**7. The first sentence of the introduction about the impact of alcoholic beverages on health is not in agreement with WHO, based on several  recent studies such as****** **Anderson BO et al, 2023 [Ref 2]**

 We agree that wine has both benefits and harmfulness depending on the consumption amount of people. We have modified the introduction stating that moderate consumption of wine can benefit human health on Lines 2-3.

**8. The word "fermentability" is used as a characteristic typical of yeast, but it would rather refer to a carbon source or a growth medium. Anyway, authors should provide an objective set of parameters defining quality of yeast in fermentation to justify the strain selection.**

 We changed the word “fermentability” into “fermentation ability” in the suitable sections of the whole study. The yeast *S. cerevisiae *2.1 is demonstrated for the quality and fermentation ability in a previous study of Ngo et al. (2005). In this study, we applied that strain of yeast for the fermentation of three-leaf trifolia.

**9. Some background about in vitro fermentation of three-leaf cayratia, their properties and biotechnological potential should be furthered commented. Otherwise, the work would remain quite out of focus.**

 Thank you for your comment. We added information on the introduction of vivo-fermentation of three-leaf trifolia wine on Lines 24-28 of Section 1 – Introduction.

**10. In section 3.1, authors state that yeast die in the highest sugar concentration condition. However,  results from a cell viability test should be provided to prove it. Anyway, another explanation would be a slower fermentation rate, probably because adjustment to higher sugar concentrations at the initial must normally requires a significant amount of time. In any case, it would be nice to have data of the dynamics of both the fermentation and the yeast growth.**

 We modified the text on Lines 99-100 which stated that “when the sugar concentration is too high, yeast fermentation is limited due to high osmotic pressure, lowering the alcoholic production”. Besides, we added one reference to clarify this point. In this test, we focus on ethanol production to assess the fermentation quality.

**11. In section 3.2, authors claim that ordinary yeast optimal temperature is 30ºC, but they probably refer to S. cerevisiae. There is a wide range of optimal growth temperatures for other yeast. Also, they state that their growth is inhibited above 32ºC, but this is simply not correct [Refer Ref 1]. After that, authors affirm that temperatures above 20ºC could provoke yeast viability to lower down, which seems contradictory. All in all, discussion of the results are not appropriated in its current state.**

 We agree with your comments. For that, we modified the text on Lines 128-130 of Section 3.2 stating that the optimal temperature for the growth and fermentation of *S. cerevisiae* is 30-35°C. We also add one more reference to clarify our explanation.
